# Histology and Ultrastructure of Transitional Changes in Skin Morphology in the Juvenile and Adult Four-Striped Mouse (*Rhabdomys pumilio*)

**DOI:** 10.1155/2013/259680

**Published:** 2013-10-28

**Authors:** Eranée Stewart, Moyosore Salihu Ajao, Amadi Ogonda Ihunwo

**Affiliations:** School of Anatomical Sciences, Faculty of Health Sciences, University of the Witwatersrand, 7 York Road, Parktown, Johannesburg 2193, South Africa

## Abstract

The four-striped mouse has a grey to brown coloured coat with four characteristic dark stripes interspersed with three lighter stripes running along its back. The histological differences in the skin of the juvenile and adult mouse were investigated by Haematoxylin and Eosin and Masson Trichrome staining, while melanocytes in the skin were studied through melanin-specific Ferro-ferricyanide staining. The ultrastructure of the juvenile skin, hair follicles, and melanocytes was also explored. In both the juvenile and adult four-striped mouse, pigment-containing cells were observed in the dermis and were homogeneously dispersed throughout this layer. Apart from these cells, the histology of the skin of the adult four-striped mouse was similar to normal mammalian skin. In the juvenile four-striped mouse, abundant hair follicles of varying sizes were observed in the dermis and hypodermis, while hair follicles of similar size were only present in the dermis of adult four-striped mouse. Ultrastructural analysis of juvenile hair follicles revealed that the arrangement and differentiation of cellular layers were typical of a mammal. This study therefore provides unique transition pattern in the four-striped mouse skin morphology different from the textbook description of the normal mammalian skin.

## 1. Introduction

The typical mammalian skin consists of an epidermis that is in contact with the free surface and an underlying dermis. Deep to the dermis is a layer of subcutaneous tissue known as the hypodermis. In mammals, skin or coat colour is essentially determined by melanocytes either situated in the epidermis or the hair bulb. These specialised cells synthesize melanin in membrane-bound organelles termed melanosomes, which are transferred through dendritic processes to their final destination [8, 12, 13, 29]. Melanocytes are specialised cells found between the stratum basale cells and in hair follicles [8–10, 12–14], although their precise distribution in mammalian skin is different from one species to another [29]. These neural crest-derived cells transfer melanosomes into surrounding cells through cytoplasmic processes [8, 12, 13, 29] and are predominantly responsible for fur, feather, and skin colour in mammals [29].

The four-striped mouse, *Rhabdomys pumilio, *is unique in that it is the only species of its genus [1] and particularly interesting where pigmentation is concerned as it has multiple dark and light stripes on its dorsal surface [2] despite all other external parts of the skin being black. This common field mouse, widely distributed in Southern Africa, has a grey to brown coloured coat with four characteristic dark stripes running along its dorsal side. The dark stripes are separated by three distinctive stripes of a lighter colour that may even be white [2]. We observed that the juvenile four-striped mouse exhibits its stripes on both the skin and coat hair, while the adult presents its stripes on the coat hair only. The histology and ultrastructure of these stripes and their transition phase for this occurrence remain to be elucidated. Our investigations provide microscopic and ultrastructural evidence of the transition in skin layers, unique pattern of the location of hair follicles, and melanocytes from juvenile to adult skin.

## 2. Materials and Methods

### 2.1. Experimental Animals

Two juvenile and two adult four-striped mice were treated and used according to the guidelines of the University of the Witwatersrand Animal Ethics Screening Committee, which parallel those set down by the National Institute of Health (NIH) for use of animals in scientific experiments. The mice originated from the Honeydew Grassland, Gauteng 27° 55′S, 26° 4′E, South Africa. A four-striped mouse with a body weight from 40 g [3] to 80 g with stripes present on both its skin and coat hair was defined as juvenile, whereas an adult was 80–95 g and having the stripes on its coat hair only. Animals were euthanised intramuscularly with 20 mg/kg ketamine after which transcardial perfusion was carried out with 0.9% saline at 4°C, followed by 4% paraformaldehyde in 0.1 M phosphate buffer [4]. The shaved skin from the dorsal striped region was harvested and processed either for light microscopy or electron microscopy.

### 2.2. Tissue Processing for Light Microscopy

Tissues were fixed in 10% buffered formalin [5] and routinely processed for light microscopy in a Shandon Citadel 1000 automatic tissue processor (UK). Paraffin wax tissue blocks were sectioned at 6 *μ*m using a Leica 1400 sledge microtome (Germany). Prepared slides were placed in an oven at 60°C for 30 minutes to ensure that the sections adhered firmly to the slides. All sections were dewaxed in 2 changes of xylene for 5 minutes each, passed through 2 changes of absolute alcohol for 30 seconds each, then transferred to 95% alcohol for 30 seconds, and rinsed in gently running tap water.

### 2.3. Haematoxylin and Eosin Staining

Sections were stained in a modified Mayer's Haematoxylin [5] for 5 minutes. Once removed from the Haematoxylin, they were left to “blue” in running tap water and staining was controlled microscopically. Sections were counterstained in Eosin for 30 seconds, followed by a brief wash in running tap water. Sections were dehydrated through a graded series of alcohol: 95% alcohol and then 2 changes of absolute alcohol. After dehydration, sections were cleared in 2 changes of xylene and mounted in Entellan.

### 2.4. Masson Trichrome Staining for Connective Tissue

Sections were stained in Iron Haematoxylin [6] for 10 minutes, washed in running tap water, differentiated in 0.5% acid alcohol, and washed thoroughly in running tap water. Sections were treated with saturated alcoholic picric acid for 3 minutes, dipped a few times in water, and stained with ponceau-fuchsin solution for 10 minutes. The sections were treated with 2% phosphotungstic acid for 5 minutes, dehydrated through a graded series of alcohols, cleared in xylene, and mounted in Entellan.

### 2.5. Ferro-Ferricyanide Staining Specific for Melanin

Sections were treated with ferrous sulfate [7] for 1 hour and washed in 4 changes of distilled water for 5 minutes each. Sections were treated with potassium ferricyanide for 30 minutes then washed in 1% glacial acetic acid for 2 minutes. Sections were counterstained in picro-ponceau for 3 to 5 minutes under microscopic control, differentiated in water, dehydrated through a graded series of alcohols, cleared in xylene, and mounted in Entellan.

### 2.6. Tissue Processing for Electron Microscopy

Small pieces of four-striped mouse skin tissue were fixed in 2.5% glutaraldehyde in phosphate buffer, pH 7.4 followed by a wash in phosphate buffer, pH 7.4 for 2 hours [4, 5]. Tissues were immersed and postfixed for 1 hour in 1% osmium tetroxide and then placed in 70% alcohol overnight in the refrigerator at 4°C. Tissues were dehydrated through 2 changes each of 95% and absolute alcohol for 20 minutes, cleared, and infiltrated with propylene oxide and Epon-Araldite resin solutions of varying ratios. First, in a solution of 3 parts propylene oxide to 1 part resin, second, in equal parts propylene oxide and resin solution, and third in a solution of 1 part propylene oxide to 3 parts resin, for a length of 30 minutes per solution. Lastly, the tissues were left overnight in resin, followed by embedding in fresh Epon-Araldite resinat 60°C for 48 hours. 

After polymerisation 1 *μ*m semithin sections was cut on a Reichert-Jung Ultracut ultramicrotome (Germany) and stained with Toluidine Blue-Pyronin Y for 30 seconds, dried, and mounted in Entellan. Semithin sections with the area of the dermis and its hair follicles, the hypodermis from the adult; the dermis and its hair follicles, the hypodermis and its hair follicles from the juvenile were selected. Ultrathin gold sections were cut and placed on copper grids and stained with uranyl acetate for 3 minutes. Drops of lead citrate were placed on strips of dental wax, and once stained, grids were rinsed first in dilute sodium hydroxide, followed by distilled water and then dried.

### 2.7. Evaluation of Slides

Light microscopy analysis of stained sections was done using a Zeiss Axioskop 2 plus Light microscope (Germany), fitted with a Zeiss Axiocam HRc camera (Germany). Ultrastructural examination and electron micrographs were taken, with a JEOL JEM-100S transmission electron microscope (Japan) at 80 kV, and negatives were scanned with an Epson Expression 1680 scanner (Japan).

## 3. Results

### 3.1. Epidermis

**Figure 1:**
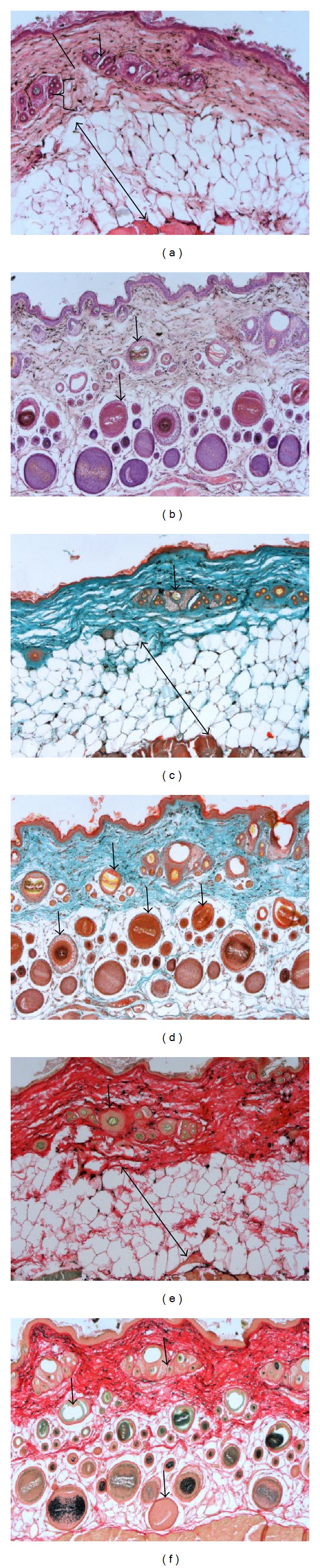
Photomicrographs of striped dorsal skin from the four-striped mouse. (a), (c), and (e) are adult skin and (b), (d), and (f) juvenile skin. Hair follicles are present in the dermis of both the adult (left) and juvenile (right). However, a marked difference is visible in the hypodermis of the juvenile containing abundant hair follicles of varying sizes (right), whereas the adult has none (left). Note the epidermis (line), dermis (bracket), hypodermis (double arrow), and hair follicles (arrows). (a) and (b) Haematoxylin and Eosin staining. (c) and (d) Masson Trichrome staining. (e) and (f) Ferro-ferricyanide staining. Scale bar (a)–(f): 10 *μ*m.

In histological studies of the adult four-striped mouse (Figures 1(a), 1(c), and 1(e)), the dermal-epidermal junction was regular with an absence of dermal papillae and epidermal ridges. Resting on a basement membrane is the stratum basale, consisting of a single layer of cuboidal to columnar cells with large, round to oval nuclei and 1 or 2 prominent nucleoli. The stratum spinosum had a thickness of 1 to 2 polyhedral cells, each with a single centrally placed oval nucleus and either 1 or 2 prominent nucleoli. The stratum granulosum appeared reduced, consisting of flattened cells with flattened oval nuclei and basophilic cytoplasmic granules (Figure 1(a)). No stratum lucidum was present. The stratum corneum consisted of several layers of extremely flattened, anucleate keratinised cells.

No melanin pigment was present in any of the epidermal layers on Ferro-ferricyanide stained sections (Figures 1(e) and 1(f)). The juvenile four-striped mouse skin (Figures 1(b), 1(d) and 1(f)) had an even dermal-epidermal junction lacking dermal papillae and epidermal ridges. The visible difference observed is that no stratum lucidum was present. The Ferro-ferricyanide stain is usually specific for any melanin pigment, usually in varying shades of green. 

### 3.2. Dermis

**Figure 2:**
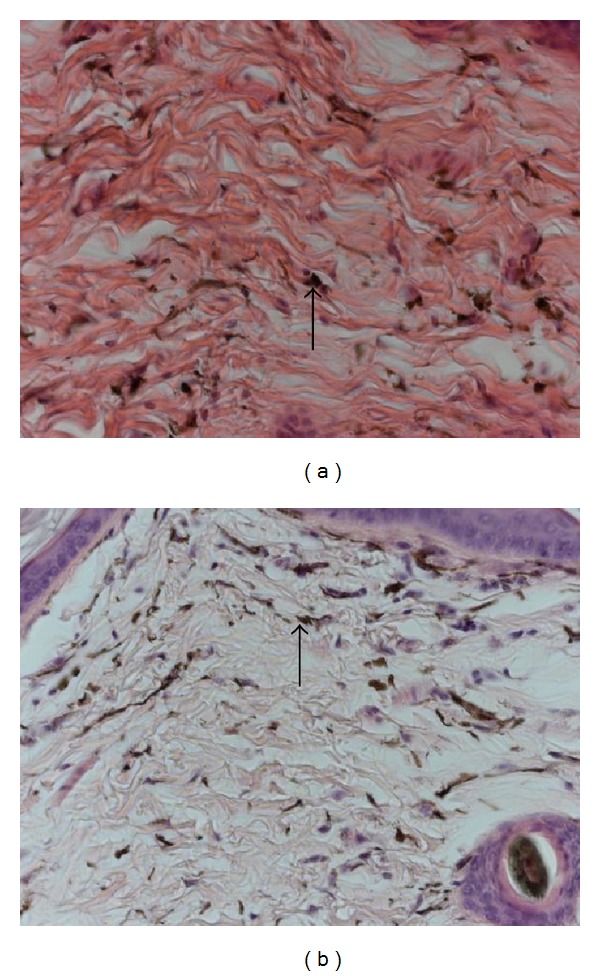
Photomicrographs of dermis from dorsal skin of the four-striped mouse. Pigment-containing cells (arrow) are homogeneously dispersed throughout the dermis. (a) Adult and (b) juvenile. Haematoxylin and Eosin, scale bar (a) and (b) = 2.5 *µ*m.

**Figure 3:**
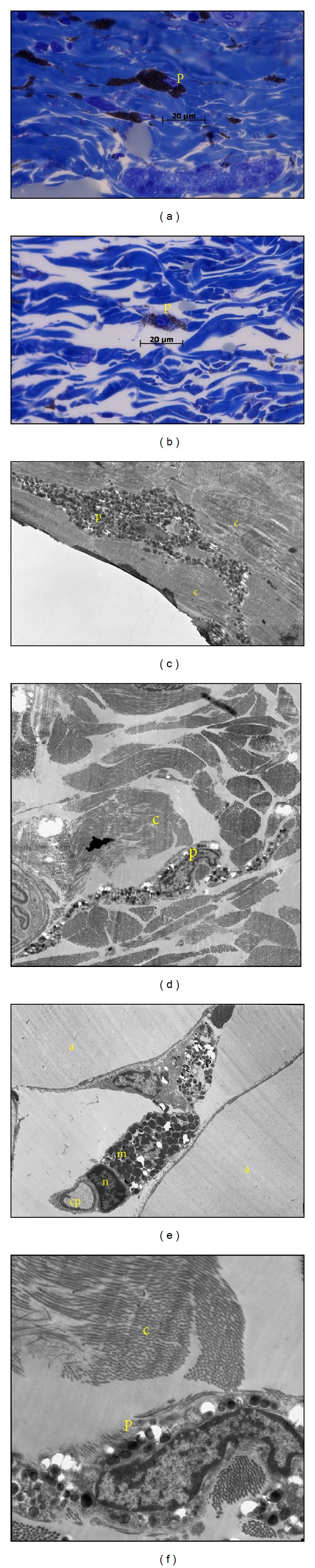
Pigment-containing cells in the four-striped mouse skin. (a), (c), and (e) from adult skin and (b), (d), and (f) from juvenile skin. Pigment-containing cell (p) is interspersed between connective tissue in (a)–(d). Pigment-containing granules are visible in the cell cytoplasm surrounding the nucleus (n) and in the long cytoplasmic extensions (e) and they also lie between adipocytes (f). (a) and (b) are photomicrographs of adult and juvenile dermis, respectively. (c) is electron micrograph of adult dermis; (d) is juvenile dermis; (e) adult hypodermis, and (f) juvenile dermis. a: adipocyte; c: collagen; cp: capillary; m: mast cell. (a) and (b) are Toluidine blue staining and scale bar = 1 *µ*m. Scale bar: (c) **=** 0.33 *µ*m; (d) **=** 0.5 *µ*m; (e) **=** 0.33 *µ*m; (f) **=** 0.17 *µ*m.

The underlying dermis of the adult skin (Figures 1(a), 1(c), and 1(e)) presented as dense irregular connective tissue with large amounts of irregularly arranged collagen fibres interspersed with fibroblasts of which only the stained nuclei were discernible. Numerous pigment-containing cells are homogeneously dispersed throughout the dermis (Figure 2(a)). These cells had large, pale-staining nuclei that were frequently obscured by large amounts of brown pigment-containing granules (Figure 3(a)).

**Figure 4:**
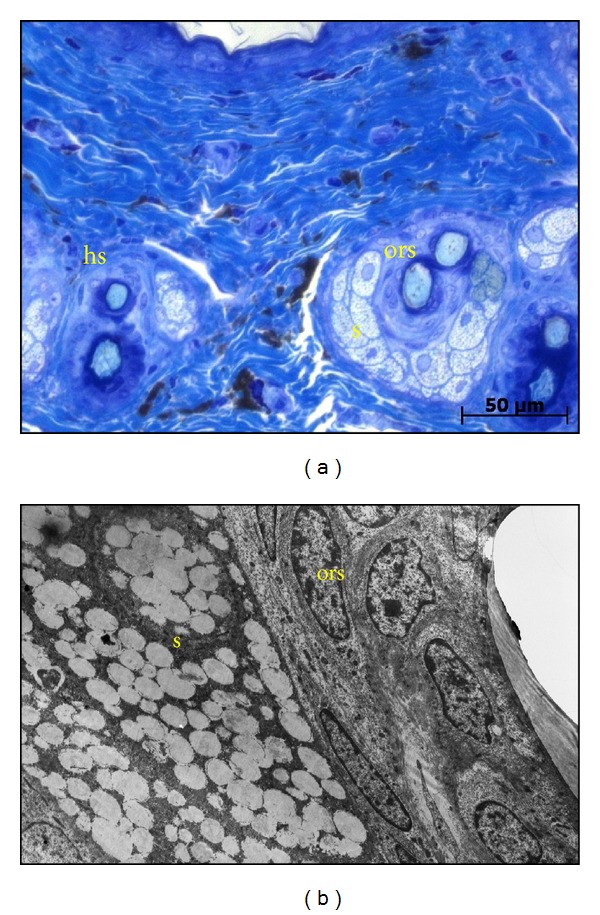
Hair follicles in the dermis of an adult four-striped mouse. Photomicrograph of multiple hair follicles (a) and electron micrograph of a single hair follicle (b). hs: hair shaft; ors: outer root sheath; s: sebaceous gland. (a) is Toluidine blue staining and scale bar = 2.5 *µ*m. (b) is an electron micrograph and with scale bar = 0.33 *µ*m.

Ultrastructurally, these cells had very long, thin cytoplasmic processes extending between collagen fibres (Figure 3(c)). Oval-shaped pigment-containing granules of varying densities were located in the cytoplasm, around the nucleus, and in the processes. Mast cells in this connective tissue layer were most noticeable in Toluidine blue sections as the cytoplasmic granules took on a purple colour due to metachromasia (Figures 3(a) and 4(a)). They were oval in shape with round to oval nuclei. An absence of eccrine sweat glands was noticed in the dermis, but hair follicles and associated simple, branched alveolar sebaceous glands were present (Figure 4(a)). The keratinised hair cortex appeared normal but a medulla was not distinct. Surrounding the hair shaft was a layer of cornified cells similar to, and continuous with, the stratum corneum. These cells continued from where the inner root sheath ended. The outer root sheath of the hair follicle in turn surrounded this.

Ultrastructurally, outer root sheath cells had oval nuclei that became progressively flattened moving laterally from the hair cortex, as the cells flattened in the same direction (Figure 4(b)). The dermis of the juvenile (Figures 1(b), 1(d), and 1(f)) was similar to that of the adult with respect to collagen, fibroblasts, mast cells and pigment-containing cells (Figures 2(b), 3(b), 3(d), and 3(f)). However, obvious differences existed in the size, location, and differentiation of juvenile hair follicles (Figures 1(b), 1(d), and 1(f)) described later. Dermal hair follicles were generally arranged in groups of mostly one large hair follicle accompanied by approximately 4 or 5 smaller ones. Larger hair follicles were generally kidney-shaped in slightly oblique sections and smaller follicles more round to oval.

### 3.3. Hypodermis

The hypodermis (Figures 1(a), 1(c), and 1(e)) was formed by a continuous unilocular adipose connective tissue layer, the panniculus adiposus. The large adipocytes were polyhedral in shape with a thin rim of cytoplasm surrounding a large unstained space within the cell and their flattened nuclei eccentrically placed. Pigment-containing cells were interspersed between the adipocytes (Figure 3(e)) and had the same morphology as those in the dermis (Figure 3(c)). Large blood vessels were located in the basal region of the hypodermis while smaller vessels (capillaries) were found throughout this layer (Figures 1(a), 1(c), and 1(e)). Mast cells were positioned in close proximity to blood vessels and between adipocytes (Figure 3(e)). Pigment-containing cells, also present in the connective tissue, surrounded the striated skeletal muscle (panniculus carnosus) beneath the hypodermis (Figures 1(a), 1(c), and 1(e)).

The juvenile hypodermis (Figures 1(b), 1(d), and 1(f)) presented the same adipose connective tissue as in the adult. In contrast to the adult, the juvenile hypodermis housed abundant hair follicles varying from small to very large in size and located throughout the entire hypodermis. Pigment-containing cells in the connective tissue surrounding the striated skeletal muscle were only occasionally present and few in numbers (Figures 1(b), 1(d), and 1(f)).

### 3.4. Melanin-Specific Staining

The Ferro-ferricyanide stain was specific for melanin on sections in varying shades of green (Figures 1(e) and 1(f)). Collagen stained red, while muscle and cytoplasm stained yellow to brown in colour. Dermal pigment-containing cells in both the juvenile and adult stained a very dark green to brown colour, proving that these cells contain melanin. Melanocytes could be identified in the hair matrix of the juvenile hair bulb (Figure 1(f)). In Ferro-ferricyanide sections of juvenile four-striped mouse skin, distinct differences were seen in the intensity of melanin staining in hair follicles (Figure 1(f)). When counted, it came to 4 regions of intensely stained hair follicles, separated by 3 regions of much lighter stained follicles. Staining intensity corresponded to the stripes seen the dorsal skin.

### 3.5. Hair Follicles in the Juvenile Skin

It is evident that the number of hair follicles visible in the section from the juvenile exceeded that in the adult. Below is a description of the hair follicles at five different levels and stages of differentiation.

#### 3.5.1. Hair Bulb and Dermal Papilla

**Figure 5:**
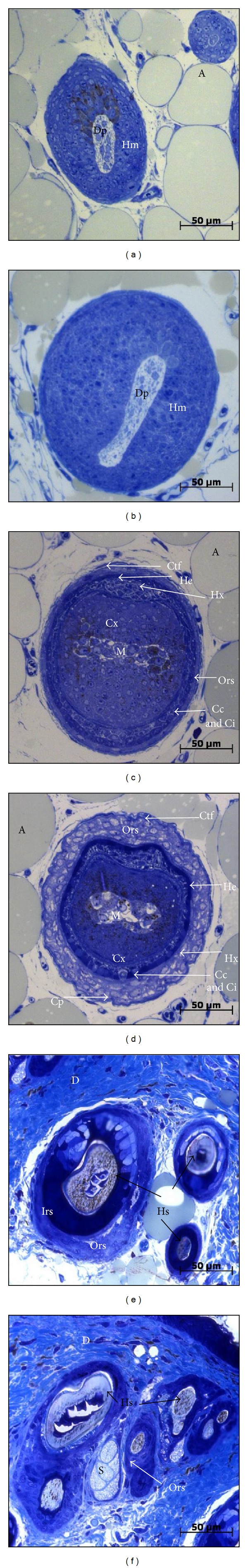
Photomicrograph of the hair follicles in the young adult four-striped mice at different levels. (a) and (b) are at the hair bulb level. (a) shows two hair follicles of small and intermediate sizes. (b) is a large follicle compared to (a). (c) shows a large follicle sectioned at the suprabulbar level, in the hypodermis. (d) is a large follicle at a level slightly higher than the suprabulbar level, in the hypodermis. (e) is follicles, in the superficial hypodermis to basal dermis. (f) shows follicles in the dermis at, or above, the sebaceous gland. A: adipocytes; Cc: cuticle of cortex; Ci: cuticle of inner root sheath; Cp: companion layer; Ctf: connective tissue follicle; Cx: cortex; D: dermis; Dp: dermal papilla; He: Henle's layer; Hm: hair matrix; Hs: hair shaft; Hx: Huxley's layer; Irs: inner root sheath; M: medulla; Ors: outer root sheath; S: sebaceous gland. Toluidine blue staining. Scale bar: 50 *µ*m.

**Figure 6:**
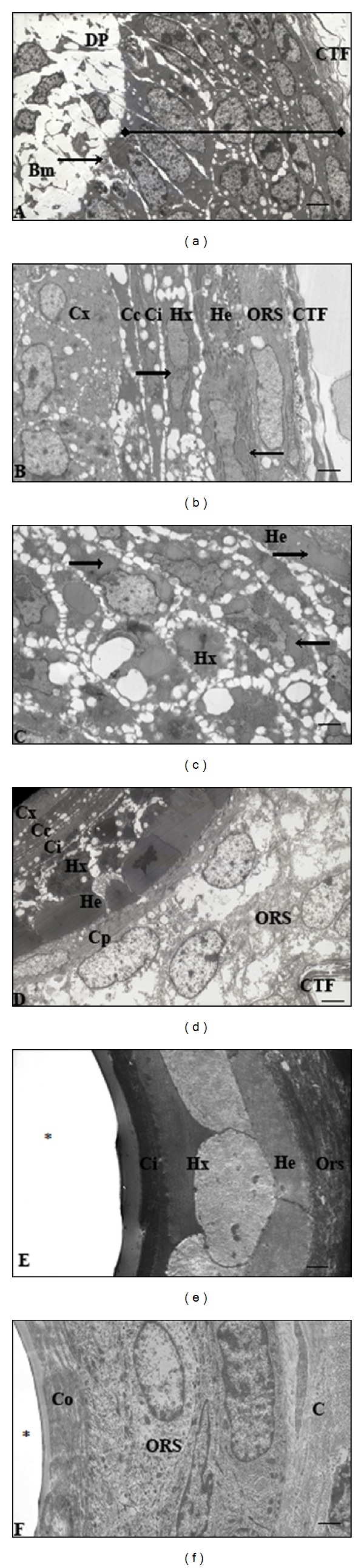
Electron micrographs of hair follicles from young adult four-striped mouse skin. (a) and (b) correspond to Figures 5(a) and 5(b) at the hair bulb and suprabulbar levels. Trichohyalin granules (arrows) are seen in the Huxley and Henle layers. (c) is at the suprabulbar level showing an accumulation of cells in Huxley's layer in the superior aspect of the follicle and corresponds to Figure 5(c). (d) is a section slightly higher than suprabulbar level and corresponds to Figure 5(d). Henle's layer and the inner root sheath are keratinised. (e) is a section of hair follicle layers at the superficial hypodermis to basal dermis and corresponds to Figure 5(e). The inner root sheath is fully keratinised in (f) above the level of the sebaceous gland and corresponds to Figure 5(f). Bm: basement membrane; C: collagen fibres; Cc: cuticle of cortex; Ci: cuticle of inner root sheath; Co: cornified cellular layer; Cp: companion layer; CTF: connective tissue follicle; Cx: cortex; DP: dermal papilla; He: Henle Layer; Hx: Huxley layer; ORS: outer root sheath; *Line*; hair matrix; *Asterisk*; area of missing hair shaft. Scale bar: (a) and (d), 0.5 *µ*m; (b) and (c), 0.33 *µ*m, (e), 0.25 *µ*m and (f), 0.20 *µ*m.

Sections at the level of the hair bulb through the dermal papilla were present throughout the hypodermis only (Figures 1(b), 1(d), and 1(f)). These hair follicles differed remarkably in size from relatively small to extremely large (Figures 5(a) and 5(b)). Ultrastructurally, these cells had very large nuclei, little cytoplasm, and thin cytoplasmic processes (Figure 6(a)). The dermal papilla was not a circular cellular column in larger follicles as is in smaller follicles (Figures 5(a) and 5(b)) but rather arranged in a wider, flattened column.

Separating the dermal papilla from the hair matrix was a basement membrane (Figure 6(a)). Follicular melanocytes were situated in the hair matrix overlying the dermal papilla. In histological sections, they contained melanosomes (Figures 1(b), 1(d), and 1(f)). On Ferro-ferricyanide sections, substantial amounts of melanin were stained green in this area (Figure 1(f)). A thin connective tissue sheath, known as the connective tissue follicle, enclosed the hair follicle separating it from the surrounding adipose tissue (Figures 5(a) and 6(a)). There was a small space between the connective tissue follicle and the outer root sheath, as they are not directly attached to each other (Figures 6(a), 6(b), and 6(d)). 

#### 3.5.2. Hair Follicle at the Suprabulbar Level

Sections of follicles at the suprabulbar level were only present in the hypodermis (Figures 1(b), 1(d), and 1(f)) and had very distinct layers surrounding the medulla (Figure 5(c)). The medulla presented the same shape as in the dermal papilla (described above) but generally not seen in small follicles. Medullary cells were large compared to cells of the surrounding layers, with the nucleus occupying a large part of the cell. Their large, round nuclei have prominent nucleoli and cells contained eosinophilic trichohyalin granules and occasionally also melanosomes in their cytoplasm (Figure 1(b)). Cells of the cortex had distinct intercellular borders, polygonal in shape with oval nuclei between 1 and 3 prominent nucleoli (Figures 5(c) and 6(c)).

Melanosomes in the cortex stained green on Ferro-ferricyanide sections (Figure 1(f)). The cuticle of the cortex and the cuticle of the inner root sheath are each formed by a single layer of thin flattened cells with flattened nuclei in both small and large follicles (Figures 5(c) and 6(b)). Surrounding the cuticle was Huxley's layer, formed by 1 to 2 layers of polygonal cells in follicles of all sizes. Nuclei had prominent nucleoli and distinct intercellular borders were visible (Figure 5(c)). Where Huxley layers had formed, the hair shaft was roughly kidney shaped. Ultrastructurally, nuclei were becoming amorphous as trichohyalin granules start to accumulate in the cytoplasm (Figure 6(c)).

Ultrastructurally, the nuclei are starting to lose their morphology as cells are filled with numerous trichohyalin granules (Figures 6(b) and 6(c)). The outer root sheath varied from a single to double layer of cells just above the hair bulb. In sections where it consisted of a double layer, more peripheral cells presented with oval shaped nuclei, while the cells in direct contact with the inner root sheath had flattened nuclei (Figures 1(b), 1(d), and 1(f)). This innermost layer is the companion layer of the outer root sheath. Lastly the connective tissue follicle (described above) surrounded the hair follicle at the periphery of the outer root sheath (Figure 5(c)).

#### 3.5.3. Hair Follicle at a Level Higher Than the Suprabulbar Region but within the Hypodermis

Near the dermal-hypodermal interface, the medulla was mostly present in relatively large follicles (Figures 1(b), 1(d), 1(f), and 5(d)). In the medulla, the cells seem to be detaching from each other but not from the cortex, forming large intercellular spaces visible under the light microscope. In the cytoplasm, the eosinophilic trichohyalin granules were decreasing, giving the medulla a clear or “glassy” appearance (Figure 1(b)). The cells of the cortex no longer had prominent intercellular borders (Figure 5(d)). In Huxley's layer, nuclear pyknosis was underway (nuclei are amorphous) and abundant trichohyalin granules, of varying sizes (Figures 5(d) and 6(d)). Keratinisation was nearly complete in both the cuticle of the inner root sheath and Henle's layer. The companion layer is morphologically distinct from the outer root sheath in both histological (Figure 5(d)) and ultrastructural analysis (Figure 6(d)). It was a single layer of flattened cells with flattened nuclei and homogeneous cytoplasm. The outer root sheath was multilayered at this level in the hair follicle (Figure 5(d)). Ultrastructurally, the cell cytoplasm exhibited electron lucent spaces (Figure 6(d)).

#### 3.5.4. Hair Follicle at the Superficial Hypodermis to Basal Dermis

In the medulla, the cell nuclei had substantially decreased in size with large intercellular spaces (Figure 5(e)). Individual cells or nuclei were no longer seen in the cortex but now filled with melanosomes. The cuticle of the cortex is a very thin transparent layer of cells adhering to the cortex with no nuclei (Figures 1(b), 5(e), and 6(e)). Trichohyalin granules were no longer present in any of the inner root sheath layers at this level (Figures 5(e) and 6(e)). The cuticle and Henle's layer were fully keratinised at this level, whereas this process is near completion in Huxley's layer. The cellular mass on the superior aspect of the hair follicle was the last of the cells from Huxley's layer to reach complete keratinisation. The outer root sheath cells had a similar appearance to the basal epidermis and stained in the same manner, as it is a continuation thereof (Figures 1(b), 1(d), and 1(f)).

#### 3.5.5. Fully Differentiated Hair Follicle at the Middle to Superficial Dermis

At this level, the medulla cortex and its cuticle were fully keratinised (Figure 5(f)). The hair shaft was completely differentiated. The inner root sheath had become continuous with the stratum corneum (forming a cornified cellular layer) above the level of the sebaceous gland and the outer root sheath is continuous with basal epithelium (Figure 6(f)). Oval nuclei of outer root sheath cells become progressively flattened towards the periphery, as the cells flatten in the same direction. At this level, the juvenile follicle (Figure 6(f)) was similar to that of the adult (Figure 4(b)).

## 4. Discussion

### 4.1. Epidermis

In human skin, melanocytes are located in the basal layer of the epidermis and the stratum basale [8–10]. From this position, they synthesize melanin for the transport to surrounding keratinocytes, which are constantly sloughed off and replaced by underlying cells. This results in continuous melanin production in the epidermal population of melanocytes [8, 11], which is the basis for human skin colour [10, 12]. An explanation for the absence of pigment in the epidermis of the four-striped mouse could be linked to the embryology of the skin in the mouse embryo as described by Hirobe [13]. Melanoblasts invade the epidermis between day 11 and 12 of gestation, complete colonisation in the epidermis by day 13 or 14, and differentiate into active melanocytes at day 16. After birth, the number of epidermal melanocytes increases dramatically, followed by migration of the melanocytes into hair bulbs where they will produce melanised hair. Because of their migration, melanocytes are only found in the epidermis for the first few weeks after birth [13] and, hence, not present in the epidermis of the juvenile and adult four-striped mouse.

### 4.2. Dermis

According to Rhodin [10], melanocytes occur occasionally in the dermis but their embryological origin remains unclear. Boissy [8] states that dermal melanocytes are not normally present in higher vertebrates but have been documented in the ears, muzzle, soles, tail, scrotum, and genital papilla of rodents. Such melanocytes contain relatively large melanosomes and have not been shown to produce additional pigment once they have been established before birth. Interestingly, melanocytes were found in the dermis of both the juvenile and adult four-striped mouse which could be associated with migration of the melanoblasts at postdifferentiation. The peculiarity of their location is reinforced by the fact that pigment-containing cells were neither present in the dermis of typical mammalian skin [14]. As these cells do not vary in pigment colouration and with their homogeneous distribution throughout the dermis, they cannot be responsible for the juvenile skin stripes. 

The pigment-containing cells in the dermis of the four-striped mouse were similar in structure to those in the naked mole-rat as reported by Daly and Buffenstein [15]. Another similarity between the two rodents is the absence of eccrine sweat glands in the dorsal skin. However, the habitats of these two rodents differ: the naked mole-rat is a strictly subterranean mammal [15], whereas the four-striped mouse lives above ground in areas that are heavily vegetated [2]. As the striped mouse also occurs in arid areas and probably originates from arid areas, this could explain why they do not have sweat glands. For a desert living animal, it might be too costly to loose water by sweating [16–18]. This could also be one of the structural adaptations to survive long periods without water drinking water as observed in laboratory based experiments with *R. pumilio* [19].

### 4.3. Hair Follicles

In the four-striped mouse, there are obvious colour differences in the hair from the striped areas due to varying pigmentation of coat hair. Juvenile hair follicles obviously outnumbered those in the adult with distinct differences in hair follicle location. Hair follicles were situated in the dermis of the adult four-striped mouse which is consistent with normal mammalian skin. In the juvenile four-striped mouse, hair follicles of different sizes were present not only in the dermis but also in the hypodermis. The location of hair follicles in the epidermis, dermis, and hypodermis in the juvenile four-stripe mouse does not conform to normal mammalian skin where hair follicles are only present in the dermis and occasionally extending into the superficial hypodermis [10]. 

The juvenile skin provided hair follicles at all levels of differentiation. Identification of tissues becomes easier as the cells differentiate while rising in the follicle [20]. The murine dermal papilla is formed by a single column of cells [21], yet it was not the case in juvenile four-striped mouse hair follicles: dermal papillae were often found to be multiple cells wide and much larger than described above. Hair bulb melanocytes were situated in the basal region of the hair matrix, resting on a basement membrane which separates it from the dermal papilla, as in a general description of the mouse by Boissy [8]. According to Niderla-Bielinska et al. [22], mesenchymal cells of the dermal papilla and dermal fibroblasts secrete regulatory factors that control the proliferation and differentiation of hair follicles. Midway up the dermal papilla undifferentiated hair matrix cells start to form 6 concentric layers from where they rise in the follicle to undergo their individual fates [21]. 

The dermal papillae in the juvenile four-striped mouse did not house a capillary as found in animals with larger hairs [21]. However, many capillaries surrounded the hair follicles in the hypodermis, possibly to supply the demand for oxygen and nutrients required by the growing hair. Slominski et al. [11] indicated that pigmented hair shafts are unique to mammals and consist of a medulla, cortex, and cuticle of the cortex. In the adult four-striped mouse follicle, no medulla was detected, yet according to Morioka [20] the hairs of the adult mouse in general contain well-developed medullae. In juvenile hair shafts, the presence of a medulla was variable. The apparent absence of a medulla in the adult four-striped mouse and the variable presence in the juvenile are possible confirmation that it is the most variable of the hair follicle tissues as postulated by Morioka [20].

The four-striped mouse medulla formed the centre core of the shaft and hardened by keratinisation similar to the inner root sheath (described later). Trichohyalin remains a major protein component of the medulla [10, 20] and stained eosinophilic as reported by these authors. The cortex forms the bulk of the hair shaft that will penetrate through the skin [20, 23]. Through differentiation, it becomes a hard, keratinized structure of the hair shaft [10] which is protected by its surrounding cuticles [23]. Generally, the cortex starts to differentiate before its cuticle [20]. The terminally differentiated cortex of the juvenile hair shaft contained no nuclei or cell organelles as cells are filled with keratin filaments [10]. In contrast to the medulla and inner root sheath, the cortex and cuticle keratinised without the presence of trichohyalin granules in accordance with the process reported [10, 20]. In the juvenile four-striped mouse, differentiation of the inner root sheath appeared normal and corresponds to the process as reported [10, 20, 21]. Trichohyalin granules appear in each of the three inner root sheath layers before keratinising [10, 20, 23, 24], as well as in the juvenile mouse. According to Alibardi et al. [24], keratinisation of the inner root sheath based on the protein trichohyalin is alike in mammals, but the process of keratinisation in the adult four-striped mouse corresponds to the description of the process in Sprague Dawley rat [20].

The inner root sheath does not form part of the hair that emerges from the skin surface, as it is released from the shaft before it projects through the skin [20]. Instead, the inner root sheath ends at the duct of the sebaceous gland, from where it joins the stratum corneum [20, 25]. At this position, both the inner root sheath and stratum corneum are shed into the follicle lumen as the hair shaft exits through the skin surface [22–24] which happened to be the same in the juvenile four-striped mouse skin. Separating the inner and outer root sheath is a monolayer of flattened cells known as the companion layer [20, 26, 27] and identified in the juvenile four-striped mouse hair follicle. In our findings, the companion layer was associated with the Henle layer instead of the outer root sheath [20, 22, 27].

In the juvenile four-striped mouse hair follicle, a single layer of cells formed the outer root sheath at the hair bulb. It thickened higher in the follicle, consisting of multiple layers of cells as reported [21, 26]. At the level of the sebaceous gland, the outer root sheath is continuous with the basal layer of epithelium [20, 22, 26, 28] as it is essentially an epidermal invagination into the dermis but cannot produce spinous, granular, or cornified cells such as the epidermis [20, 22]. The presence of a specialised area of the outer root sheath known as the bulge area, located just below the sebaceous gland [22, 29], has been reported by some investigators as not only providing attachment for the arrector pili muscle but also the location of hiding multipotent stem cells [20, 22, 28, 29]. The basal layer of epithelium migrates to contribute to the maintenance of epithelium and epithelial derivatives including hair follicles and sebaceous glands [20, 22, 29]. The hair follicle of the juvenile four-striped mouse was encircled by a connective tissue sheath that consisted of several layers of collagen fibres and fibroblasts, as reported for the Sprague Dawley rat [20].

## 5. Conclusion

In general, the layers of the skin from both the adult and juvenile four-striped mice conformed to the structure of normal mammalian skin. The only exception was the pigment-containing cells located in dermis of the four-striped mouse. The morphology of the epidermis as well as dermal pigment-containing cells was the same in both the juvenile and adult four-striped mouse. The epidermis lacked pigmentation, while the melanocytes were homogeneously distributed throughout the dermis. Hair follicles were present in the dermis and the hypodermis of the juvenile but confined to dermis in the adult. The major and unique differences observed between the juvenile and adult four-striped mouse were the size, structure, and location of the hair follicles. Further ultrastructural analysis of juvenile hair follicles established that their structure and differentiation were similar to normal mammalian follicles. Although little is known about the underlying reasons for the structural differences the description provided can form the basis for further investigation especially of the biology of the transition process of the hair follicles.
